# Subjective health complaints are not associated with tick bites or antibodies to *Borrelia burgdorferi* sensu lato in blood donors in western Norway: a cross-sectional study

**DOI:** 10.1186/s12889-015-2026-5

**Published:** 2015-07-14

**Authors:** Reidar Hjetland, Harald Reiso, Camilla Ihlebæk, Roy M. Nilsen, Nils Grude, Elling Ulvestad

**Affiliations:** Department of Microbiology, Førde General Hospital, Helse Førde Hospital Trust, PO Box 1000, NO-6807 Førde, Norway; Institute of Health and Society, Department of General Practice, University of Oslo, Oslo, Norway; Section of public health, ILP, Norwegian University of Life Sciences, Aas, Norway; Centre for Clinical Research, Haukeland University Hospital, Bergen, Norway; Department of Microbiology, Vestfold Hospital Trust, Tønsberg, Norway; Department of Microbiology, Haukeland University Hospital, Bergen, Norway; Department of Clinical Science, University of Bergen, Bergen, Norway

**Keywords:** Lyme disease, Lyme borreliosis, Blood donors, Subjective health complaints, Tick bites

## Abstract

**Background:**

There is controversy about chronic health consequences of tick-borne infections, especially Lyme borreliosis. This study aims to assess whether general function, physical fitness and subjective health complaints are associated with tick bites or antibodies to *Borrelia burgdorferi* sensu lato in blood donors.

**Methods:**

Sera from 1,213 blood donors at four different blood banks in Sogn and Fjordane county in western Norway were obtained during January to June 2010, and analysed for specific IgG and IgM antibodies. A questionnaire including questions on tick bites, subjective health complaints, general function and physical fitness was completed.

**Results:**

Tick bites had been experienced by 65.7 % of the study population. 78 (6.4 %) were positive for IgG (9.7 % in men, 2.4 % in women), and 69 (5.7 %) for IgM (6.1 % in men, 5.1 % in women), verified by immunoblot. No association between number of experienced tick bites or seropositivity for Borrelia antibodies and subjective health complaints, reduced general function or reduced physical fitness was found.

**Conclusion:**

The results do not support any association between tick bites or Borrelia antibodies and subjective health complaints in blood donors in an endemic area for Lyme borreliosis.

**Electronic supplementary material:**

The online version of this article (doi:10.1186/s12889-015-2026-5) contains supplementary material, which is available to authorized users.

## Background

There is much controversy about chronic health consequences of treated and untreated Lyme borreliosis (LB) [1–4]. Symptoms from many organ systems are ascribed to “chronic LB”, including fatigue, stiff neck, migrating arthralgias, myalgia, chest pain, palpitations, abdominal pain, nausea, back pain, headaches, etc. [3]. In addition, other agents transmitted by ticks, e.g., *Rickettsia* spp., are increasingly claimed to cause health problems [5].

In follow-up studies of patients with Lyme neuroborreliosis (LNB), several symptoms have been found to persist in some of the patients compared to controls, including malaise, fatigue, memory problems, concentration difficulties and paresthesias [6, 7].

Population based follow-up studies of patients treated for LB in general have given discrepant results regarding long-term consequences. Some American studies have reported that LB patients experience long-lasting symptoms [8, 9], while other investigations found that symptoms at follow-up did not exceed that of a control population [10–14].

European studies have also shown that patients having suffered from early LB report a number of symptoms; however, these did not exceed the corresponding symptoms in matched controls [15, 16].

In a recent Swedish study, patients bitten by *Borrelia*-infected ticks were compared to patients bitten by non-infected ticks [17]. The frequency of subjects reporting symptoms was higher in the group bitten by *Borrelia*-infected ticks compared to subjects bitten by uninfected ticks. There were, however, no differences between subjects bitten by a *Borrelia*-positive tick and subjects bitten by a *Borrelia*-negative tick when comparing the frequency of reported experience of several symptoms, such as fatigue, myalgia/arthralgia, headache, and neck pain.

It thus seems that long-lasting symptoms after LNB are frequent in adults; however, in properly controlled studies, lasting symptoms are rarely seen after early disease manifestations such as erythema migrans or after bites with *Borrelia*-infected ticks.

A majority of blood donors in Sogn and Fjordane county have experienced tick bites [18], and the seroprevalence of antibodies to *B. burgdorferi* s.l. is substantial [19]. The aim of this study was to assess the association, if any, of general function, physical fitness and subjective health complaints with reported tick bites and antibodies to *B. burgdorferi* sensu lato in this group of healthy adults.

## Methods

### Study population

During the period January 13th to June 15th 2010, blood donors at the four blood banks in Sogn and Fjordane were asked to participate in the Tick-borne Infection Study in Sogn and Fjordane. A total of 1,213 blood donors participated, a response rate of 76 %. The mean age was 45.8 years, ranging from 19 to 69. Men constituted 55.2 %. Further characteristics of the participants are presented elsewhere [18]. Informed consent was obtained from each participant, and the study was approved by the Regional Committee for Medical Research Ethics. This investigation included a questionnaire in addition to testing serum samples for antibodies to *B. burgdorferi* s.l., tick-borne encephalitis virus and *Anaplasma phagocytophilum*. The reported occurrence of tick bites and the results of the antibody tests in this study population have been published elsewhere [18–20].

### Questionnaire

The questionnaire included questions about demographics such as gender, age, marital status, education, household income and occupation. Questions on pet animals, farm animals, hours spent outdoors during summertime, hunting, orienteering, smoking, and symptoms and treatment after tick bites were also included.

The questionnaire included two questions on tick bites; tick bites ever experienced, and tick bites experienced during the last 12 months. The responses for both questions were given in the categories “none”, “one”, “2-5”, “6-20” and “more than 20”. The number of tick bites ever experienced was used in the present study.

Another section of the questionnaire included the Subjective Health Complaints (SHC) Inventory, a set of questions designed to measure common and prevalent health complaints in the general population [21–23]. The respondents reported to what extent they had been affected by 29 different health disturbances during the last month. The response options were “Not at all” (score 0), “A little” (score 1), “Some” (score 2), and “Serious” (score 3). In addition, the respondents were asked to record the duration of these symptoms (1–30 days).

The question related to general function was “How do you assess your ability to perform ordinary activity, your general function, is today?” The response options were “Good, as it usually is” (score 0), “Hardly reduced at all” (score 1), “Not much reduced” (score 2), “Moderately reduced” (score 3) and “Much reduced” (score 4), adapted from [24].

Physical fitness was assessed by the question “«During the past 2 weeks: What was the hardest physical activity you could do for at least 2 min?», with the response alternatives «Very heavy (ex. run, at fast pace)» (score 0), « Heavy (ex. jog, at slow pace)» (score 1), « Moderate (ex. walk, at a fast pace)» (score 2), «Light (ex. walk, at a medium pace)» (score 3), and «Very light (ex. walk, at a slow pace or not able to walk)» (score 4). This question was taken from the COOP/WONCA charts [25].

The questionnaires were answered anonymously, and most were completed during the time spent at the blood bank for routine donation.

### Laboratory methods

For testing of antibodies to *B. burgdorferi* s.l., all sera were tested in Enzygnost Lyme link VlsE/IgG and Enzygnost Borreliosis IgM (DADE Behring, Marburg, Germany) and in Immunetics C6 Lyme ELISA kit (Immunetics, Cambridge, MA, USA). Sera showing positive or grey-zone reactivities in any of these tests were further tested in Borrelia-EUROLine-RN-AT IgG and Borrelia-EUROLine-RN-AT IgM (Euroimmun AG, Lübeck, Germany) immunoblots. Further details are presented elsewhere [19]. The results of the immunoblots are used in the present study, and are here-after referred to as IgG and IgM.

### Risk factors

The three risk factors included in the present analyses were the number of self-reported tick bites ever experienced, positive IgG, and positive IgM for *B. burgdorferi* sensu lato. With the exeption of the questions on general function and physical fitness (Table 1), the numbers of tick bites were dichotomised into less than two versus two or more in the analyses.

**Table 1:** Overall function and physical fitness in relation to tick bites and Borrelia antibodies

Risk factor		Reduced overall function		Reduced physical fitness	
		Odds ratio^2^ (95 % CI)	Adjusted odds ratio^3^ (95 % CI)	Odds ratio^2^ (95 % CI)	Adjusted odds ratio^3^ (95 % CI)
Total tick bites ever experienced					
n^1^		1167	1145	1179	1157
None (%)	34.1	1	1	1	1
1 (%)	18.1	1.9 (1.0 - 3.5)	1.7 (0.9 - 3.2)	1.2 (0.9 - 1.6)	1.1 (0.8 - 1.5)
2-5 (%)	23.7	0.7 (0.4 - 1.5)	0.7 (0.4 - 1.6)	0.8 (0.6 - 1.0)	0.7 (0.5 - 0.9)
6-20 (%)	15	1. 9 (1.0 - 3.5)	1.8 (0.9 - 3.5)	0.6 (0.5 - 0.9)	0.5 (0.4 - 0.7)
>20 (%)	9.2	2.1 (1.0 - 4.4)	2.0 (0.9 - 4.2)	0.9 (0.6 - 1.3)	0.7 (0.4 - 1.0)
p trend		0.086	0.117	0.012	<0.001
IgG					
n^1^		1179	1157	1191	1169
negative (%)	93.8	1	1	1	1
positive (%)	6.2	1.6 (0.8 - 3.5)	1.7 (0.8 - 3.7)	1.1 (0.7 - 1.7)	1.1 (0.7 - 1.7)
p difference		0.216	0.204	0.725	0.688
IgM					
n^1^		1179	1157	1191	1169
negative (%)	94.3	1	1	1	1
positive (%)	5.7	1.5 (0.7 - 3.5)	1.5 (0.7 -3.4)	1.1 (0.7 - 1.7)	0.9 (0.6 - 1.5)
p difference		0.296	0.340	0.760	0.726

^1^Numbers do not add to 1,213 due to missing data

^2^Odds ratio was estimated using proportional odds ratio models (logistic regression model with more than two categorical outcomes)

^3^Adjusted for gender, age group and blood bank

### Outcome

The outcome variables in this study were the responses to questions on subjective health complaints (SHC), general function, and physical fitness.

For general function and physical fitness, the response was categorized in the categories 0–3, i.e., categories 3 and 4 were merged, because of few respondents in category 4.

For the SHC-questions, we calculated the total number of complaints reported by each participant. In addition, a total SHC score was computed as the sum of the severity scores of all 29 items in the questionnaire, the computation of this score was allowed if 18 or more of the questions were answered.

The proportion of subjects reporting any complaint within five subscales are reported as well; these include “musculoskeletal” complaints (headache, neck pain, shoulder pain, pain in arms, pain in upper back, low back pain, leg pain, and pain in the feet during exercise), “pseudoneurological” complaints (extra heartbeats, heat flushes, sleep problems, tiredness, dizziness, anxiety and sadness/depression), “gastrointestinal” complaints (heartburn, stomach discomfort, ulcer/non-ulcer dyspepsia, stomach pain, bloating, diarrhoea and constipation), “allergic” complaints (asthma, breathing difficulties, eczema, allergies and chest pain) and “flu” (cold/flu and cough). These subscales are based on factor analysis and the factors have shown good reliability in previous studies [21]. The computation of these proportions allowed for a certain number of missing values, which were ignored if constituting less than half of the questions within the subscale.

For the statistical evaluation of each individual SHC we chose to dichotomize all responses into “no complaint” versus any degree of complaint, as very few scored the two most serious responses 2 and 3.

### Statistical analysis

We used IBM SPSS Statistics version 21 (SPSS Inc., Chicago, Illinois) for statistical analyses except for Table 3, where Stata/SE version 13.1 for Windows (StataCorp LP, College Station, Texas) was used.

Immunoblot results for IgG and IgM were categorized as positive or negative, with grey-zone results included as negatives.

For the questions on general function and physical fitness, the association between the score and number of self-reported tick bites was analysed by using proportional odds models, i.e., logistic regression models with more than two ordinal categorical outcomes, with and without adjustment for gender, age group and location of blood bank [26].

For SHC, both individually for each complaint as well as for the five sub-scales, the associations between the risk factors and outcome variables were analysed by binary logistic regression, with and without adjustment for gender, age group and location of blood bank.

Differences in the total number of complaints and the total SHC score between groups with and without the risk factors were estimated by using linear regression model with robust variance estimation. All p values were two-sided and values below 0.05 were considered statistical significant.

## Results

Among participants, 65.7 % had experienced tick bites during their life time. One tick bite was reported by 18.0 %, 2–5 bites by 23.8 %, 6–20 by 14.7 %, and more than 20 bites by 9.2 %. The estimated mean number of tick bites was 5.7, as published elsewhere [18].

Borrelia IgG antibodies verified by immunoblot were present in 6.4 % (9.7 % in men and 2,4 % in women), whereas Borrelia IgM antibodies were demonstrated in 5.7 % (6.1 % in men and 5.1 % in women) [19].

Overall, 5.0 % reported to have received antibiotics because of tick bite or consequences thereof. Among IgG and/or IgM blot positives, antibiotics had been received by 11.9 %.

The results for the relationship between the risk factors tick bites ever experienced, *Borrelia* IgG and *Borrelia* IgM, and the outcomes general function and physical fitness are presented in Table 1. The proportion reporting reduced general function did not differ significantly between subjects with and without the risk factors. The number of tick bites was positively associated with good physical fitness (adjusted p for trend < 0.001).

The relationship between the proportion reporting any complaint within the five subscales of subjective health complaints and the risk factors are presented in Table 2, and results for each of the individual 29 complaints are presented in the Additional file 1.

**Table 2:** Odds ratio for reporting any degree of subjective health complaint (SHC), grouped in five subscales

Complaint/Risk factor (RF)	n^1^	Proportion with complaint (%)		Odds ratio^2^ (OR) for complaint when risk factor present			
		RF absent	RF present	Unadjusted	p^3^	Adjusted^4^	p^3^
Musculoskeletal pain							
Tick bites >1	1139	66.6	68.1	1.1 (0.8 - 1.4)	0.583	1.1 (0.8 - 1.4)	0.578
IgG +	1151	67.1	69.0	1.1 (0.6 - 1.8)	0.743	1.3 (0.7 - 2.2)	0.374
IgM +	1151	66.9	72.6	1.3 (0.7 - 2.3)	0.359	1.5 (0.8 - 2.7)	0.172
Pseudoneurology							
Tick bites >1	1136	40.8	39.2	0.9 (0.7 - 1.2)	0.570	0.9 (0.7 - 1.1)	0.264
IgG +	1148	41.1	23.9	0.5 (0.3 - 0.8)	0.005	0.5 (0.3 - 0.8)	0.010
IgM +	1148	40.2	37.1	0.9 (0.5 - 1.5)	0.624	0.8 (0.5 - 1.5)	0.522
Gastrointestinal complaints							
Tick bites >1	1136	41.5	42.1	1.0 (0.8 - 1.3)	0.829	1.0 (0.8 - 1.3)	0.991
IgG +	1148	42.0	38.0	0.8 (0.5 - 1.4)	0.515	0.9 (0.5 - 1.4)	0.557
IgM +	1148	41.4	46.8	1.2 (0.7 - 2.1)	0.408	1.1 (0.7 - 1.9)	0.632
Allergy							
Tick bites >1	1136	20.2	19.8	1.0 (0.7 - 1.3)	0.870	0.9 (0.7 - 1.2)	0.534
IgG +	1148	20.2	16.9	0.8 (0.4 - 1.5)	0.497	0.8 (0.4 - 1.5)	0.415
IgM +	1148	19.7	25.8	1.4 (0.8 - 2.6)	0.245	1.4 (0.7 - 2.5)	0.324
Flu							
Tick bites >1	1161	29.3	31.4	1.1 (0.9 - 1.4)	0.434	1.2 (0.9 - 1.5)	0.264
IgG +	1174	29.7	36.5	1.4 (0.8 - 2.2)	0.222	1.3 (0.8 - 2.2)	0.273
IgM +	1174	29.5	42.2	1.7 (1.0 - 2.9)	0.033	1.3 (0.8 - 2.2)	0.273

^1^n does not reach 1213 because of missing data

^2^Odds ratio for subjects without the risk factor is 1

^3^Binary logistic regression

^4^Adjusted for gender, age group and blood bank location

There were no significant associations between the proportion reporting “musculoskeletal pain” or the individual related complaints with any of the risk factors.

For the group of “pseudoneurological” complaints, there were fewer complaints in the subjects with *Borrelia* IgG than in those without these antibodies (adjusted *p* = 0.010). None of the individual complaints in this group of questions were significantly associated with any of the risk factors. However, for all questions there were fewer in the IgG positive group reporting symptoms than among IgG negatives (Additional file 1).

In the subscale “gastrointestinal” complaints, some more subjects with more than one tick bite reported ulcer- and non-ulcer dyspepsia (adjusted *p* = 0.079), and there was a trend towards more IgM positives reporting constipation (Additional file 1; adjusted *p* = 0.081).

There were no significant associations between the number of subjects reporting complaints within the “allergy” subscale or the individual related complaints with any of the risk factors.

The “flu”-related questions all showed a higher percentage of IgM positives reporting symptoms, however, this associations were weakened when adjusted for gender, age group and blood bank location.

We found no significant associations between the total number of subjective health complaints or the total SHC score and any of the risk factors (Table 3) when adjusted for gender, age, and location of blood bank.

**Table 3:** Number of subjective health complaints and total SHC score relative to tick bites and Borrelia antibodies

	No. subjects^1^	Median (IQR)^2^	Mean (SEM)^3^	Adjusted mean (SEM)^3,4^
**Number of complaints**				
Tick-bites				
≤1	624	3.0 (1.0-5.0)	3.59 (0.14)	3.62 (0.13)
>1	570	3.0 (1.0-5.0)	3.64 (0.13)	3.60 (0.14)
p difference			0.785	0.903
IgG				
-	1135	3.0 (1.0-5.0)	3.62 (0.10)	3.63 (0.10)
+	78	2.0 (1.0-4.0)	3.29 (0.50)	3.42 (0.51)
p difference			0.527	0.603
IgM				
-	1144	3.0 (1.0-5.0)	3.58 (0.10)	3.60 (0.10)
+	69	3.0 (1.0-5.0)	3.94 (0.51)	3.87 (0.51)
p difference			0.480	0.600
**Total SCH score** ^**5**^				
Tick-bites				
≤1	595	3.0 (1.0-6.0)	4.71 (0.24)	4.76 (0.25)
>1	541	3.1 (1.0-6.0)	4.58 (0.20)	4.51 (0.20)
p difference			0.672	0.440
IgG				
-	1077	3.0 (1.0, 6.0)	4.62 (0.14)	4.60 (0.14)
+	71	3.0 (1.0, 5.0)	5.22 (1.31)	5.36 (1.26)
p difference			0.644	0.550
IgM				
-	1086	3.0 (1.0-6.0)	4.57 (0.14)	4.57 (0.14)
+	62	3.1 (2.0-7.0)	6.06 (1.41)	5.94 (1.35)
p difference			0.295	0.313

^1^The number of subjects does not reach 1213 because of missing data

^2^Interquartile range (IQR)

^3^Standard error of the mean (SEM) predicted by linear regression with robust variance estimation

^4^Adjusted for gender, age and blood bank location

^5^SCH: Subjective health complaints. Total SHC score: See text

The additional questions on duration of the different symptoms were answered by only 60 % (38-73 %) of the subjects reporting any degree of the complaints. Therefore, these data were not considered satisfactory for statistical evaluation and are not shown.

## Discussion

The main finding in this study was the lack of association between the risk factors tick bites and Borrelia antibodies with the outcomes subjective health complaints, reduced general function and reduced physical fitness. On the contrary, we found a positive association between tick bites and good physical fitness, and between presence of *Borrelia* IgG and low occurrence of “pseudoneurological” complaints. For most complaints, however, we found no association.

The risk factor “number of tick bites” was included in this study to explore the possibility that seronegative LB or other tick-borne infectious agents not tested for, e.g. *Anaplasma phagocytophilum*, *Rickettsia* spp., etc., could give rise to chronic health problems of some magnitude.

IgG and IgM antibodies to *B. burgdorferi* s.l. verified by blot were chosen as risk factors in this study in order to avoid including false positive ELISA results. However, biologically false positive IgM blot results still are a problem, and American guidelines thus argue against using IgM blot in the second-tier testing when disease duration is longer than one month [27]. A proportion of the IgM blot positive specimens in this study are probably biologically false positive, as discussed elsewhere [19]. Also, many will lose their antibodies after a while, whether treated or not. However, antibodies against *B. burgdorferi* s.l., especially IgG, should be an indicator of former or present infection with this organism.

In several studies of symptoms after LB, the Medical Outcomes Study 36-Item Short-Form Health Survey (SF-36) has been used [6, 8–10]. In addition, several questionnaires for assessing somatic symptoms in the general population exist [28]. The unique contribution of the current study was to include the SHC questionnaire, which was designed to measure common and prevalent health complaints in the general population, and has been used in several studies of the general population as well as different patients groups [29–32].

Compared to other surveys using the SHC Inventory in the general population, summarised by Eriksen et al. in 1999 [21], and for a working population more recently by Ihlebæk and co-workers [29], the proportion of subjects with any complaint were lower in our material for most complaints, reflecting the overall healthy status of the blood donors (Additional file 1).

Comparing our results to those of other studies is not straight forward, of two main reasons. Firstly, the selection of study population differs between studies, e.g., blood donors, subjects having been bitten by ticks, patients having suffered from LB, patients having suffered from LNB, etc. Secondly, the choice of questionnaire also varies. We are not aware of other studies using the SHC inventory in blood donors or in relation to LB. Nevertheless, in the following, a tentative comparison is attempted.

In a study of 1156 male military recruits in Germany, Treib and co-workers found that *Borrelia* IgG positive subjects reported significantly more fatigue, general malaise and limb pain, compared to seronegatives [33]. This is in contrast to our findings, where musculoskeletal pain in different locations as well as “tiredness”, “reduced general function” and “reduced physical fitness” was reported as often by seronegatives as by seropositives for IgG.

Studies by Shadic and co-workers from 1994 and 1999 found that persons with a history of LB had more musculoskeletal impairment and a higher prevalence of verbal memory impairment when compared with those without a history of LB [8, 9]. As these reports are based on a different study population as well as a different questionnaire, they are not directly comparable with our present study. However, our results do not corroborate these findings, as musculoskeletal pain and reduced general function were not found associated with seropositivity or number of tick bites.

In agreement with our results, Seltzer and co-workers [10] reported that persons having suffered from LB generally did not report more symptoms than controls, including memory problems, numbness, fatigue, swollen joints, headaches, neck pain, or problems with sleeping and exercise. Also, supporting our findings, in two recent Slovenian studies of patients treated for erythema migrans, the researchers did not find any association between cases and controls in non-specific symptoms 6 months after treatment [15, 16]. In long-term follow-up of patients with culture-confirmed LB, Wormser and co-workers did not find evidence of severe fatigue or fibromyalgia attributable to LB [11, 12], and summary scores of physical and mental health were similar to those of the general population [14].

As part of the Swedish “STING-study”, Fryland and co-workers [17] compared symptoms in patients bitten by ticks infected with *Borrelia burgdorferi* sensu lato with patients bitten with non-infected ticks. They found a higher frequency of patients reporting any symptom in the former group, but no differences between the groups when comparing the frequency of each of several symptoms. In the present study, we found that the count of subjective health complaints had no relation with any of the risk factors (Table 3).

In a prospective investigation of patients treated for LNB in Norway, Eikeland and co-workers found that LNB-treated patients scored lower on all the SF-36 subscores except on bodily pain, and they reported fatigue, memory problems and concentration difficulties more often than matched controls [6]. Notable was that in contrast to the results from Shadic et al., pain was not more common among LNB-treated patients than among controls, neither assessed by direct question nor by SF-36. These data are not comparable to our study, as very few, if any, of our study subjects have suffered from LNB [18].

According to alternative views on LB, there are many undiagnosed patients with nonspecific chronic symptoms attributable to the disease [3]. Among the listed symptoms, some are included in the SHC inventory. The prevalence of these was not significantly higher for any of the risk factors tick bites, IgG or IgM in our study. However, a number of symptoms are not explicitly included in the SHC inventory. If these symptoms were of any significance in our study population, it should be reflected in the score on “reduced general function” in subjects with the risk factors. This was, however, not the case.

The clear correlation between the number of tick bites and physical fitness is not surprising. Persons more exposed to ticks are presumably more involved in outdoors activities such as hiking and hunting, and are in a generally good physical condition.

The negative association of “pseudoneurological” complaints and Borrelia IgG antibodies, but not number of tick bites or IgM, is difficult to explain. Also here, a spurious relationship connected to confounding lifestyle factors can be suspected.

The strength of this study is the relatively large representation of healthy subjects from Sogn and Fjordane county, the good response rate, and the frequent occurrence of tick bites and seropositivity to *B. burgdorferi* s.l., as well as the scope of the questionnaire, covering a broad range of health complaints. Thus, major chronic health effects of tick bites and seropositivity to *B. burgdorferi* s.l. should be detected.

On the other hand, blood donors are not completely representative of the general population. They are healthy, and children and persons over 70 years are not represented. There was, however, a fair distribution regarding gender and age groups. According to Norwegian blood bank regulations, persons that have been bitten by ticks should not donate blood within four weeks of the bite, and persons with suspected or verified LB should not donate until six months after adequate treatment has been given [34]. Donors with recent tick bites and/or LB may therefore be underrepresented. Not all tick bites are recognized by the bitten person, and thus will not be reported in a questionnaire survey like this (i.e., information bias). This is supported by the seroprevalence rate for immunoblot verified IgG antibodies to *B. burgdorferi* s.l. of 2.4 % in subjects not reporting any tick bite [19].

It is to be expected that persons with significant chronic health problems do not volunteer as blood donors, or will be excluded. Persons of this category are therefore probably underrepresented in this material. However, given the commonness of these “soft” symptoms also in blood donors (Table 2), one would expect that mild degrees of such complaints should be represented.

## Conclusions

This study of blood donors in western Norway assessed the association between three risk factors – the number of tick bites ever experienced and the presence of Borrelia IgG and IgM antibodies – with the questionnaire-based outcomes general function, physical fitness and 29 different subjective health complaints.

A limitation of this study is that very few of the study subjects reported ever having had symptoms indicative of systemic LB.

There was no association between general function and the risk factors. However, we found a clear correlation between the number of tick bites ever experienced and good physical fitness. This most probably represents a spurious relationship as both risk factor and outcome are related to a healthy life style.

We did not find any association between more subjective health complaints and tick bites or Borrelia antibodies. Seeing the «pseudoneurological» group of complaints together, there were fewer complaints in subjects with Borrelia IgG antibodies, also probably a non-causal relationship.

The results do not support any association between tick bites or Borrelia antibodies and subjective health complaints in blood donors in an endemic area for Lyme borreliosis.

## Additional file

Additional file 1:
**Individual subjective health complaints relative to tick bites and Borrelia antibodies.**
