# Supplementary material for: Subjective health complaints are not associated with tick bites or antibodies to Borrelia burgdorferi sensu lato in blood donors in western Norway: a cross-sectional study
Source: BMC Public Health. 2015 Jul 14;15:657. doi: 10.1186/s12889-015-2026-5 (PMC4499943; doi:10.1186/s12889-015-2026-5)
Supplement: Additional file 1: — Individual subjective health complaints relative to tick bites and Borrelia antibodies. [file 12889_2015_2026_MOESM1_ESM.docx]

**Additional file Individual subjective health complaints relative to tick bites and Borrelia antibodies**

| Complaint/  risk factor (RF) | n^1^ | Propor­tion with RF (%) |  | Propor­tion with complaint (%) | |  | Odds ratio (OR) for complaint when risk factor present (binary logistic regression) | | | | |
| --- | --- | --- | --- | --- | --- | --- | --- | --- | --- | --- | --- |
|  |  |  |  | RF ab­sent | RF pre­sent |  | Unadjusted | p |  | Adjusted^2^ | p |
| Headache |  |  |  |  |  |  |  |  |  |  |  |
| Tick bites >1 | 1148 | 47.8 |  | 36.4 | 34.2 |  | 0.9 (0.7 - 1.2) | 0.447 |  | 1.0 (0.8 - 1.3) | 0.841 |
| IgG + | 1159 | 6.1 |  | 35.7 | 31.0 |  | 0.8 (0.5 - 1.4) | 0.425 |  | 1.2 (0.7 - 2.1) | 0.469 |
| IgM + | 1159 | 5.3 |  | 35.7 | 29.0 |  | 0.7 (0.4 - 1.3) | 0.285 |  | 0.9 (0.5 - 1.6) | 0.726 |
| Neck pain |  |  |  |  |  |  |  |  |  |  |  |
| Tick bites >1 | 1137 | 47.8 |  | 25.9 | 25.6 |  | 1.0 (0.8 - 1.3) | 0.900 |  | 1.0 (0.7 - 1.3) | 0.949 |
| IgG + | 1149 | 6.2 |  | 26.1 | 21.1 |  | 0.8 (0.4 - 1.4) | 0.358 |  | 0.8 (0.5 - 1.5) | 0.572 |
| IgM + | 1149 | 5.5 |  | 25.8 | 25.4 |  | 1.0 (0.5 - 1.8) | 0.946 |  | 1.0 (0.5 - 1.8) | 0.974 |
| Upper back pain |  |  |  |  |  |  |  |  |  |  |  |
| Tick bites >1 | 1121 | 47.5 |  | 10.4 | 13.1 |  | 1.3 (0.9 - 1.9) | 0.152 |  | 1.2 (0.8 - 1.8) | 0.325 |
| IgG + | 1133 | 6.2 |  | 11.8 | 11.4 |  | 1.0 (0.5 - 2.1) | 0.934 |  | 1.0 (0.4 - 2.1) | 0.953 |
| IgM + | 1133 | 5.6 |  | 11.7 | 12.7 |  | 1.1 (0.5 - 2.4) | 0.808 |  | 1.0 (0.5 - 2.3) | 0.934 |
| Lower back pain |  |  |  |  |  |  |  |  |  |  |  |
| Tick bites >1 | 1137 | 47.6 |  | 30.9 | 32.7 |  | 1.1 (0.8 - 1.4) | 0.505 |  | 1.0 (0.8 - 1.4) | 0.726 |
| IgG + | 1149 | 6.1 |  | 31.5 | 34.3 |  | 1.1 (0.7 - 1.9) | 0.629 |  | 1.1 (0.7 - 1.9) | 0.679 |
| IgM + | 1149 | 5.3 |  | 32.1 | 24.6 |  | 0.7 (0.4 - 1.3) | 0.224 |  | 0.7 (0.4 - 1.3) | 0.287 |
| Arm pain |  |  |  |  |  |  |  |  |  |  |  |
| Tick bites >1 | 1127 | 47.4 |  | 17.2 | 16.1 |  | 0.9 (0.7 - 1.3) | 0.622 |  | 0.8 (0.6 - 1.1) | 0.178 |
| IgG + | 1139 | 6.1 |  | 16.6 | 15.9 |  | 1.0 (0.5 - 1.8) | 0.881 |  | 0.8 (0.4 - 1.6) | 0.572 |
| IgM + | 1139 | 5.4 |  | 16.2 | 24.2 |  | 1.7 (0.9 - 3.0) | 0.101 |  | 1.5 (0.8 - 2.9) | 0.172 |
| Shoulder pain |  |  |  |  |  |  |  |  |  |  |  |
| Tick bites >1 | 1143 | 48.0 |  | 27.3 | 30.2 |  | 1.2 (0.9 - 1.5) | 0.269 |  | 1.0 (0.8 - 1.4) | 0.792 |
| IgG + | 1155 | 6.1 |  | 28.6 | 28.6 |  | 1.0 (0.6 - 1.7) | 1.000 |  | 1.0 (0.5 - 1.7) | 0.877 |
| IgM + | 1155 | 5.3 |  | 28.1 | 37.7 |  | 1.6 (0.9 - 2.6) | 0.107 |  | 1.5 (0.9 - 2.6) | 0.129 |
| Migraine |  |  |  |  |  |  |  |  |  |  |  |
| Tick bites >1 | 1126 | 47.5 |  | 6.3 | 8.0 |  | 1.3 (0.8 - 2.1) | 0.248 |  | 1.2 (0.7 - 1.9) | 0.447 |
| IgG + | 1138 | 6.2 |  | 7.0 | 7.0 |  | 1.0 (0.4 - 2.6) | 0.997 |  | 1.1 (0.4 - 2.9) | 0.864 |
| IgM + | 1138 | 5.4 |  | 7.0 | 8.2 |  | 1.2 (0.5 - 3.1) | 0.714 |  | 1.2 (0.5 - 3.3) | 0.651 |
| Pain in feet after strain |  |  |  |  |  |  |  |  |  |  |  |
| Tick bites >1 | 1138 | 47.8 |  | 9.9 | 11.2 |  | 1.1 (0.8 - 1.7) | 0.483 |  | 1.1 (0.7 - 1.6) | 0.754 |
| IgG + | 1150 | 6.3 |  | 10.3 | 13.9 |  | 1.4 (0.7 - 2.8) | 0.338 |  | 1.3 (0.6 - 2.8) | 0.433 |
| IgM + | 1150 | 5.4 |  | 10.3 | 14.5 |  | 1.5 (0.7 - 3.1) | 0.295 |  | 1.4 (0.7 - 3.0) | 0.376 |
| Palpitations |  |  |  |  |  |  |  |  |  |  |  |
| Tick bites >1 | 1136 | 47.6 |  | 7.6 | 8.7 |  | 1.2 (0.8 - 1.8) | 0.488 |  | 1.1 (0.7 - 1.8) | 0.566 |
| IgG + | 1148 | 6.1 |  | 8.4 | 2.9 |  | 0.3 (0.1 - 1.3) | 0.115 |  | 0.3 (0.1 - 1.4) | 0.140 |
| IgM + | 1148 | 5.4 |  | 7.8 | 12.9 |  | 1.7 (0.8 - 3.8) | 0.159 |  | 1.7 (0.8 - 3.7) | 0.200 |
| Heat flushes |  |  |  |  |  |  |  |  |  |  |  |
| Tick bites >1 | 1138 | 47.8 |  | 10.1 | 10.5 |  | 1.0 (0.7 - 1.5) | 0.834 |  | 0.7 (0.4 - 1.0) | 0.075 |
| IgG + | 1150 | 6.1 |  | 10.5 | 8.6 |  | 0.8 (0.3 - 1.9) | 0.615 |  | 1.2 (0.5 - 3.3) | 0.665 |
| IgM + | 1150 | 5.4 |  | 10.3 | 10.3 |  | 1.1 (0.5 - 2.5) | 0.802 |  | 1.0 (0.4 - 2.6) | 0.973 |
| Sleep problems |  |  |  |  |  |  |  |  |  |  |  |
| Tick bites >1 | 1137 | 47.8 |  | 17.7 | 16.0 |  | 0.9 (0.7 - 1.2) | 0.457 |  | 0.8 (0.6 - 1.1) | 0.142 |
| IgG + | 1149 | 6.2 |  | 17.3 | 11.3 |  | 0.6 (0.3 - 1.3) | 0.191 |  | 0.6 (0.3 - 1.3) | 0.208 |
| IgM + | 1149 | 5.4 |  | 17.3 | 11.3 |  | 0.6 (0.3 - 1.4) | 0.225 |  | 0.6 (0.3 - 1.3) | 0.203 |
| Tiredness |  |  |  |  |  |  |  |  |  |  |  |
| Tick bites >1 | 1140 | 47.8 |  | 24.7 | 25.7 |  | 1.1 (0.8 - 1.4) | 0.703 |  | 1.0 (0.8 - 1.4) | 0.826 |
| IgG + | 1152 | 6.2 |  | 25.8 | 15.5 |  | 0.5 (0.3 - 1.0) | 0.056 |  | 0.6 (0.3 - 1.1) | 0.103 |
| IgM + | 1152 | 5.3 |  | 25.1 | 25.8 |  | 1.0 (0.6 - 1.9) | 0.906 |  | 1.0 (0.6 - 1.9) | 0.957 |
| Dizziness |  |  |  |  |  |  |  |  |  |  |  |
| Tick bites >1 | 1135 | 47.8 |  | 7.1 | 6.1 |  | 0.9 (0.5 - 1.4) | 0.501 |  | 0.8 (0.5 - 1.3) | 0.427 |
| IgG + | 1147 | 6.2 |  | 6.8 | 5.6 |  | 0.8 (0.3 - 2.3) | 0.708 |  | 0.8 (0.3 - 2.4) | 0.702 |
| IgM + | 1147 | 5.4 |  | 6.5 | 9.7 |  | 1.5 (0.6 - 3.7) | 0.341 |  | 1.6 (0.6 - 3.9) | 0.313 |
| Anxiety |  |  |  |  |  |  |  |  |  |  |  |
| Tick bites >1 | 1138 | 47.6 |  | 3.9 | 2.6 |  | 0.7 (0.3 - 1.3) | 0.228 |  | 0.6 (0.3 - 1.2) | 0.148 |
| IgG + | 1150 | 6.2 |  | 3.3 | 2.8 |  | 0.8 (0.2 - 3.6) | 0.813 |  | 0.8 (0.2 - 3.5) | 0.759 |
| IgM + | 1150 | 5.5 |  | 3.2 | 3.2 |  | 1.5 (0.4 - 5.0) | 0.508 |  | 1.4 (0.4 - 4.6) | 0.628 |
| Depressed |  |  |  |  |  |  |  |  |  |  |  |
| Tick bites >1 | 1136 | 47.6 |  | 6.2 | 6.3 |  | 1.0 (0.6 - 1.6) | 0.963 |  | 1.0 (0.6 - 1.7) | 0.881 |
| IgG + | 1148 | 6.2 |  | 6.3 | 4.2 |  | 0.7 (0.2 - 2.1) | 0.482 |  | 0.7 (0.2 - 2.5) | 0.609 |
| IgM + | 1148 | 5.4 |  | 6.0 | 9.7 |  | 1.7 (0.7 - 4.1) | 0.245 |  | 1.7 (0.7 - 4.1) | 0.252 |
| Heartburn |  |  |  |  |  |  |  |  |  |  |  |
| Tick bites >1 | 1144 | 47.6 |  | 19.0 | 17.8 |  | 0.9 (0.7 - 1.2) | 0.611 |  | 0.9 (0.6 - 1.2) | 0.369 |
| IgG + | 1156 | 6.2 |  | 18.2 | 23.6 |  | 1.4 (0.8 - 2.4) | 0.252 |  | 1.2 (0.7 - 2.1) | 0.589 |
| IgM + | 1156 | 5.4 |  | 18.1 | 25.4 |  | 1.5 (0.9 - 2.8) | 0.151 |  | 1.3 (0.7 - 2.5) | 0.343 |
| Stomach discomfort |  |  |  |  |  |  |  |  |  |  |  |
| Tick bites >1 | 1134 | 47.4 |  | 4.9 | 5.4 |  | 1.1 (0.7 - 1.9) | 0.689 |  | 1.0 (0.6 - 1.7) | 0.918 |
| IgG + | 1146 | 6.2 |  | 5.3 | 4.2 |  | 0.8 (0.2 - 2.6) | 0.694 |  | 0.6 (0.2 - 2.1) | 0.423 |
| IgM + | 1146 | 5.4 |  | 5.4 | 3.2 |  | 0.6 (0.1 - 2.5) | 0.470 |  | 0.5 (0.1 - 2.1) | 0.353 |
| Ulcer and non-ulcer dyspepsia |  |  |  |  |  |  |  |  |  |  |  |
| Tick bites >1 | 1134 | 47.7 |  | 0.5 | 1.7 |  | 3.3 (0.9 - 12.4) | 0.073 |  | 3.6 (0.9 - 14.8) | 0.079 |
| IgG + | 1146 | 6.2 |  | 0.9 | 4.2 |  | 4.7 (1.3 - 17.5) | 0.021 |  | 3.0 (0.7 - 12.7) | 0.140 |
| IgM + | 1146 | 5.4 |  | 7.8 | 6.6 |  | 3.3 (0.7 - 15.0) | 0.131 |  | 3.3 (0.6 - 16.9) | 0.159 |
| Stomach pain |  |  |  |  |  |  |  |  |  |  |  |
| Tick bites >1 | 1132 | 47.6 |  | 7.1 | 8.2 |  | 1.2 (0.8 - 1.8) | 0.493 |  | 1.2 (0.8 - 2.0) | 0.357 |
| IgG + | 1144 | 6.1 |  | 7.5 | 10.0 |  | 1.4 (0.6 - 3.1) | 0.456 |  | 1.7 (0.7 - 3.9) | 0.248 |
| IgM + | 1144 | 5.3 |  | 7.8 | 6.6 |  | 0.8 (0.3 - 2.4) | 0.733 |  | 0.9 (0.3 - 2.6) | 0.835 |
| Bloating |  |  |  |  |  |  |  |  |  |  |  |
| Tick bites >1 | 1142 | 47.6 |  | 24.9 | 23.7 |  | 0.9 (0.7 - 1.2) | 0.636 |  | 0.9 (0.7 - 1.2) | 0.375 |
| IgG + | 1154 | 6.2 |  | 24.5 | 22.2 |  | 0.9 (0.5 - 1.6) | 0.664 |  | 0.9 (0.5 - 1.6) | 0.688 |
| IgM + | 1154 | 5.5 |  | 24.4 | 23.8 |  | 1.0 (0.5 - 1.8) | 0.918 |  | 0.8 (0.4 - 1.5) | 0.456 |
| Diarrhoea |  |  |  |  |  |  |  |  |  |  |  |
| Tick bites >1 | 1136 | 47.7 |  | 10.3 | 10.5 |  | 1.0 (0.7 - 1.5) | 0.891 |  | 1.1 (0.7 - 1.6) | 0.688 |
| IgG + | 1148 | 6.2 |  | 10.3 | 12.7 |  | 1.3 (0.6 - 2.6) | 0.528 |  | 1.3 (0.6 - 2.8) | 0.450 |
| IgM + | 1148 | 5.4 |  | 10.7 | 6.5 |  | 0.6 (0.2 - 1.6) | 0.296 |  | 0.6 (0.2 - 1.6) | 0.302 |
| Constipation |  |  |  |  |  |  |  |  |  |  |  |
| Tick bites >1 | 1133 | 47.7 |  | 3.4 | 4.1 |  | 1.2 (0.7 - 2.2) | 0.541 |  | 1.2 (0.6 - 2.4) | 0.518 |
| IgG + | 1145 | 6.1 |  | 3.7 | 4.3 |  | 1.2 (0.3 - 3.8) | 0.810 |  | 1.4 (0.4 - 4.9) | 0.623 |
| IgM + | 1145 | 5.3 |  | 3.5 | 8.2 |  | 2.5 (0.9 - 6.5) | 0.069 |  | 2.5 (0.9 - 6.8) | 0.081 |
| Asthma |  |  |  |  |  |  |  |  |  |  |  |
| Tick bites >1 | 1133 | 47.7 |  | 3.5 | 2.0 |  | 0.6 (0.3 - 1.2) | 0.132 |  | 0.5 (0.2 - 1.1) | 0.089 |
| IgG + | 1145 | 6.1 |  | 2.9 | 2.9 |  | 1.0 (0.2 - 4.2) | 0.990 |  | 0.9 (0.2 - 3.9) | 0.879 |
| IgM + | 1145 | 5.4 |  | 2.7 | 6.5 |  | 2.5 (0.9 - 7.4) | 0.095 |  | 2.3 (0.8 - 7.0) | 0.130 |
| Breathing difficulties |  |  |  |  |  |  |  |  |  |  |  |
| Tick bites >1 | 1135 | 47.6 |  | 2.0 | 2.0 |  | 0.8 (0.3 - 2.0) | 0.662 |  | 0.7 (0.3 - 1.7) | 0.453 |
| IgG + | 1147 | 6.2 |  | 1.9 | 2.8 |  | 1.5 (0.4 - 6.7) | 0.571 |  | 1.0 (0.2 - 4.4) | 0.962 |
| IgM + | 1147 | 5.4 |  | 1.8 | 4.8 |  | 2.9 (0.8 - 9.9) | 0.099 |  | 1.9 (0.5 - 7.0) | 0.310 |
| Eczema |  |  |  |  |  |  |  |  |  |  |  |
| Tick bites >1 | 1137 | 47.6 |  | 8.7 | 10.2 |  | 1.2 (0.8 - 1.8) | 0.406 |  | 1.1 (0.7 - 1.6) | 0.693 |
| IgG + | 1149 | 6.1 |  | 9.7 | 4.3 |  | 0.4 (0.1 - 1.3) | 0.142 |  | 0.4 (0.1 - 1.2) | 0.104 |
| IgM + | 1149 | 5.4 |  | 9.2 | 12.9 |  | 1.5 (0.7 - 3.2) | 0.334 |  | 1.4 (0.6 - 3.1) | 0.394 |
| Allergies |  |  |  |  |  |  |  |  |  |  |  |
| Tick bites >1 | 1141 | 47.8 |  | 9.6 | 8.4 |  | 0.9 (0.6 - 1.3) | 0.509 |  | 0.9 (0.6 - 1.4) | 0.590 |
| IgG + | 1153 | 6.2 |  | 9.1 | 8.5 |  | 0.9 (0.4 - 2.2) | 0.843 |  | 1.1 (0.4 - 2.7) | 0.851 |
| IgM + | 1153 | 5.4 |  | 8.8 | 14.5 |  | 1.8 (0.8 - 3.7) | 0.133 |  | 1.8 (0.9 - 3.9) | 0.114 |
| Chest pain |  |  |  |  |  |  |  |  |  |  |  |
| Tick bites >1 | 1135 | 47.6 |  | 2.9 | 2.6 |  | 0.9 (0.4 - 1.9) | 0.785 |  | 0.7 (0.3 - 1.5) | 0.381 |
| IgG + | 1147 | 6.2 |  | 2.6 | 5.6 |  | 2.2 (0.8 - 6.6) | 0.143 |  | 1.5 (0.5 - 4.5) | 0.504 |
| IgM + | 1147 | 5.4 |  | 2.6 | 6.5 |  | 2.6 (0.9 - 7.7) | 0.083 |  | 2.0 (0.7 - 6.3) | 0.211 |
| Cold, flu |  |  |  |  |  |  |  |  |  |  |  |
| Tick bites >1 | 1152 | 47.9 |  | 25.8 | 26.6 |  | 1.0 (0.8 - 1.4) | 0.759 |  | 1.1 (0.8 - 1.4) | 0.504 |
| IgG + | 1165 | 6.3 |  | 25.6 | 32.9 |  | 1.4 (0.9 - 2.4) | 0.175 |  | 1.4 (0.8 - 2.4) | 0.181 |
| IgM + | 1165 | 5.4 |  | 25.6 | 34.9 |  | 1.6 (0.9 - 2.7) | 0.103 |  | 1.5 (0.9 - 2.6) | 0.153 |
| Cough |  |  |  |  |  |  |  |  |  |  |  |
| Tick bites >1 | 1140 | 47.8 |  | 11.9 | 11.7 |  | 1.0 (0.7 - 1.4) | 0.921 |  | 1.0 (0.7 - 1.4) | 0.929 |
| IgG + | 1151 | 6.3 |  | 11.9 | 9.7 |  | 0.8 (0.4 - 1.8) | 0.585 |  | 0.7 (0.3 - 1.7) | 0.493 |
| IgM + | 1151 | 5.6 |  | 11.3 | 18.8 |  | 1.8 (0.9 - 3.5) | 0.076 |  | 1.5 (0.8 - 3.0) | 0.239 |

^1^ n does not reach 1213 because of missing data

^2^ Adjusted for gender, age group and blood bank location
